# An RNA-binding compound that stabilizes the HIV-1 gRNA packaging signal structure and specifically blocks HIV-1 RNA encapsidation

**DOI:** 10.1186/s12977-018-0407-4

**Published:** 2018-03-14

**Authors:** Carin K. Ingemarsdotter, Jingwei Zeng, Ziqi Long, Andrew M.L. Lever, Julia C. Kenyon

**Affiliations:** 1 Department of Medicine, University of Cambridge, Addenbrookes Hospital, Box 157, Level 5, Hills Rd, Cambridge, CB2 0QQ UK; 2 Department of Medicine, National University of Singapore, Singapore, Singapore; 3 Department of Microbiology and Immunology, National University of Singapore, Singapore, Singapore; 4 Homerton College, University of Cambridge, Cambridge, UK

**Keywords:** HIV-1, Antiretroviral drugs, Packaging, RNA structure

## Abstract

**Background:**

NSC260594, a quinolinium derivative from the NCI diversity set II compound library, was previously identified in a target-based assay as an inhibitor of the interaction between the HIV-1 (ψ) stem-loop 3 (SL3) RNA and Gag. This compound was shown to exhibit potent antiviral activity. Here, the effects of this compound on individual stages of the viral lifecycle were examined by qRT-PCR, ELISA and Western blot, to see if its actions were specific to the viral packaging stage. The structural effects of NSC260594 binding to the HIV-1 gRNA were also examined by SHAPE and dimerization assays.

**Results:**

Treatment of cells with NSC260594 did not reduce the number of integration events of incoming virus, and treatment of virus producing cells did not affect the level of intracellular Gag protein or viral particle release as determined by immunoblot. However, NSC260594 reduced the incorporation of gRNA into virions by up to 82%, without affecting levels of gRNA inside the cell. This reduction in packaging correlated closely with the reduction in infectivity of the released viral particles. To establish the structural effects of NSC260594 on the HIV-1 gRNA, we performed SHAPE analyses to pinpoint RNA structural changes. NSC260594 had a stabilizing effect on the wild type RNA that was not confined to SL3, but that was propagated across the structure. A packaging mutant lacking SL3 did not show this effect.

**Conclusions:**

NSC260594 acts as a specific inhibitor of HIV-1 RNA packaging. No other viral functions are affected. Its action involves preventing the interaction of Gag with SL3 by stabilizing this small RNA stem-loop which then leads to stabilization of the global packaging signal region (psi or ψ). This confirms data, previously only shown in analyses of isolated SL3 oligonucleotides, that SL3 is structurally labile in the presence of Gag and that this is critical for the complete psi region to be able to adopt different conformations. Since replication is otherwise unaffected by NSC260594 the flexibility of SL3 appears to be a unique requirement for genome encapsidation and identifies this process as a highly specific drug target. This study is proof of principle that development of a new class of antiretroviral drugs that specifically target viral packaging by binding to the viral genomic RNA is achievable.

**Electronic supplementary material:**

The online version of this article (10.1186/s12977-018-0407-4) contains supplementary material, which is available to authorized users.

## Background

For HIV there is now a range of effective drugs targeting different stages of viral replication yet mutational escape from all of these has been achieved by the virus. Between the stages of integration of the provirus and virion maturation however there is a therapeutic gap wherein many of the processes are not virus specific. Exceptions include compounds targeting viral assembly binding to the HIV-1 Gag protein, or its NC domain, and interfering with genome encapsidation or virion maturation [1–3]. Others work by destabilizing and ejecting zinc from the two zinc knuckles in the NC domain. Almost all of them target the viral protein [4, 5].

The HIV-1 5′UTR is around 330 nucleotides (nt) long and contains a series of overlapping *cis*-acting signals that interact with viral and cellular factors to control the viral lifecycle. It is far more highly conserved than the coding regions that follow it, with little or no variation observed between hundreds of sequenced clinical isolates in some sections [6]. This degree of conservation against the background of a high viral mutation rate indicates the essential nature of these sequences, the structures they subtend and the inability of the virus to tolerate mutation within them. Compounds that target the Gag-RNA interaction by binding to highly conserved sequences of the RNA instead of the cognate protein may provide far less scope for mutational escape. One such region that shows extremely limited variation is a 14 nt stretch just upstream of the Gag translation initiation site. This RNA folds into a stable helix known as SL3, which has been shown to be of importance during the viral packaging process, by specifically binding the Gag protein in a high-affinity interaction [7, 8].

NSC260594 (hereafter referred to as NSC) is a quinolinium derivative that we identified in a high-throughput screen for compounds that interfere with the interaction between SL3 and Gag [9]. By NMR analysis this compound was shown to bind specifically to the terminal loop of SL3 where Gag is known to interact. NSC was also shown to inhibit production of infectious HIV in vitro at micromolar concentrations. The SL3 sequence has few predicted counterparts in the human genome, and the compound displays low cytotoxicity. This suggested that within infected cells its effect is related to it inhibiting genome encapsidation although this was not formally shown.

However, despite circumstantial evidence that NSC binds to SL3 and interferes with Gag binding in vitro, the HIV-1 5′UTR contains multiple *cis*- and *trans*-acting functions, including other protein binding sites and there is evidence that it undergoes a range of conformational changes in order to facilitate viral replication [10]. It is therefore possible that a compound that binds to SL3 exerts its antiviral effects at other and/or multiple stages of the HIV lifecycle. NSC has also been shown in a recent screen to be a protein binding inhibitor of the dengue virus 2 protease [11].

To clarify the true effect of NSC in a replicating viral system and to attempt to validate the potential for development of RNA targeting antiretrovirals, and antipackaging inhibitors, we examined the specific role of NSC in inhibiting multiple stages of the HIV-1 lifecycle. We show that NSC specifically targets the viral packaging process, resulting in significantly lower presence of genomic viral RNA inside the virions. It does not affect the steady state levels of viral unspliced genomic RNA (gRNA) or Gag protein inside cells. It does not interfere with viral reverse transcription or integration. When assessed by immunoblot we find that NSC does not significantly reduce viral particle production.

To further evaluate the mechanism by which NSC interferes with gRNA encapsidation the structural changes induced by NSC binding to the viral leader were studied by SHAPE chemistry. We show that NSC binding to SL3 has effects on several parts of the leader RNA, including SL3 itself. These changes were not observed when NSC was incubated with an SL3 deleted RNA. These findings suggest that NSC disrupts the interaction of SL3 with Gag but also, through stabilizing SL3, it leads to reduced conformational flexibility of the wider backbone structure of the RNA in the 5′leader. These observations reveal the critical nature of structural change in the RNA during the encapsidation process and provide proof-of-principle that specific RNA-targeting anti-packaging compounds are an achievable therapeutic aim.

## Methods

### Reagents

NSC260594 (Benzamide, 4-[(1-methyl-6-nitro-4(1H)-quinolinylidene)amino]-*N*-[4-[(1-methyl-4(1H)-pyridinylidene)amino]phenyl]-, molecular weight 505.0) was supplied by NCI-DTP (www.dtp.cancer.gov). It was dissolved in DMSO at 20 mM and stored at − 20 °C. Plasmids pSVC21ΔBglII [12] pSVC21ΔBglIIΔp1 [13, 14] pVSVG [15] and pBluescript were grown in *E. coli* DH5α strain and purified using Maxiprep columns (Qiagen), and used to produce HIVΔEnv (WT) and HIVΔEnvΔpI (ΔpI) viruses. The ΔpI mutation is a 19-nt deletion of the SL3 region. Jurkat cells were maintained in RPMI 1640 medium (Gibco, Life Technologies) supplemented with 10% fetal bovine serum (Gibco, Life Technologies), penicillin [100 U/ml] and streptomycin [100 µg/ml] (Gibco, Life Technologies). 293T and TZMbl cells were maintained in DMEM containing 10% (v/v) FCS, 100 U/mL penicillin and 100 μg/mL streptomycin (w/v). TZMbl is a reporter cell line derived from HeLa cells expressing CXCR4, engineered to express CD4 and CCR5 [16], the firefly luciferase gene and the *E. coli* β-galactosidase gene under the control of the HIV-1 LTR [16, 17]. It was obtained from the NIH AIDS Research and Reference Reagent Program. Full-length LAI virus was produced by transfection of pLAI, an infectious molecular clone of HIV-1 LAI, into 293T cells for 48 h and the supernatant harvested and cleared of cell debris by centrifugation at 3488×*g* for 5 min. An LAI plasmid-free virus stock was produced by infecting Jurkat cells with virus-containing supernatant followed by serial passaging in Jurkat cells. The LAI containing supernatant was cleared of cell debris by centrifugation at 2671×*g* for 5 min prior to the integration assay described below.

### qRT-PCR to measure intracellular and extracellular HIV RNA levels

293T cells were plated 24 h before transfection in six-well plates at a density of 10^6^ cells/well. Transfections were performed with TransIT-LT1 (Mirus) using 150 ng of either pSVC21ΔBglII or pSVC21ΔBglIIΔp1 and 50 ng of pVSVG and following manufacturer’s instructions. pBluescript up to 1 μg was added to all wells as a bulking agent for transfection and to ensure even amounts of DNA were added to each well. 6 h post transfection cells were treated with NSC dissolved in DMSO to a final concentration of 50 μM or an equal volume of DMSO only. 24 h post treatment, supernatants and cellular fractions were collected. RNA was extracted from cells using the Qiagen RNeasy kit according to manufacturer’s instructions, but without DNase treatment on the columns. DNA was subsequently removed by DNase treatment of a smaller volume of the purified nucleic acid with TURBO DNase (Thermo Fisher Scientific) in DNase buffer for 2 h at 37 °C and the RNA was recovered with phenol–chloroform extraction and ethanol precipitation. Supernatants were harvested for purification of RNA from virions. Initially supernatants were clarified at 5600×*g* for 10 min to pellet cell debris. Virions were then purified by centrifuging a 2:1 ratio of supernatant: 8.4% Optiprep in PBS at 21,500×*g* for 90 min. Virions were resuspended in 10% of the original supernatant volume of PBS followed by the addition of 10× that volume of Proteinase K buffer (50 mM Tris–Cl pH 7.5, 100 mM NaCl, 10 mM EDTA, 1% SDS, 100 μg/ml proteinase K, 100 μg/ml yeast tRNA) and incubation for 30 min at 37 °C. RNA was recovered with phenol–chloroform extraction and ethanol precipitation, resuspended in 1× DNase buffer and treated with 1/10th volume of TURBODNase (Thermo Fisher Scientific) for 60 min at 37 °C, phenol–chloroform extracted and ethanol precipitated once more and resuspended in water.

### Cytoplasmic RNA extraction

293T cells were transfected and treated with NSC as described above. Cytoplasmic RNA was extracted with the RNeasy Mini kit (Qiagen) following the manufacturer’s supplementary protocol with minor modifications. In brief, 24 h post-NSC treatment, 293T cells were washed in PBS followed by cell lysis with 175 µl chilled RLN buffer (50 mM Tris–Cl, pH8, 140 mM NaCl, 1.5 mM MgCl_2_, 0.5% NP-40 (v/v)). Cells were removed by scraping and incubated on ice for 5 min, followed by centrifugation for 2 min at 300×*g*. The supernatant was removed and mixed with 600 µl RLT buffer (Qiagen), followed by incubation for 15 min at room temperature then vortexed. Samples were temporarily stored at − 80 °C, and 430 µl 96–100% ethanol was added to each sample prior to loading of samples onto RNeasy spin columns according to the manufacturer’s instructions without on-column DNase digestion. The RNA was eluted twice in 30 µl nuclease free H_2_0. Extracted RNA was subjected to DNase treatment with the Turbo DNA-*free*™ Kit (Thermo Fisher Scientific). In brief, 1 µg RNA was treated with 1 µl TURBO DNase in 5 µl 10× TURBO DNase Buffer in a total volume of 50 µl for 1 h at 37 °C. After 1 h, 5 µl DNase inactivation reagent was added to each reaction and incubated at room temperature for 5 min. The reactions were spun at 10,000×*g* for 1.5 min and the supernatant containing DNase-treated RNA was transferred to a fresh tube.

### HIV *gag* qRT-PCR

DNase-treated RNA was reverse transcribed with the High Capacity cDNA Reverse Transcription Kit (Thermo Fisher Scientific) according to the manufacturer’s instructions with RNase inhibitor added to the reaction, or with the addition of RNasin^®^ Ribonuclease Inhibitor (Promega). The PCR cycling conditions were; 25 °C for 10 min, 37 °C for 120 min, 85 °C for 5 min followed by a holding step at 4 °C. The cDNA was further diluted to 1–4 ng and subjected to HIV *gag* qPCR with 25–50 nM HIV *gag* forward primer; HIV *gag* 6F: 5′-CATGTTTTCAGCATTATCAGAAGGA-3′, and 25–50 nM HIV *gag* reverse primer; HIV *gag* 84R: 5′-TGCTTGATGTCCCCCCACT-3′ and 100 nM HIV gag probe: 5′-[6FAM]CCACCCCACAAGATTTAAACACCATGCTAA[BHQ1]-3′ (Sigma) [18] in a reaction buffer containing 2× Taqman Fast Advanced Master Mix, (Thermo Fisher Scientific). The HIV *gag* qPCR conditions were as follows: Step 1. 50 °C for 2 min. Step 2, 95 °C for 20 s. Step 3. 95 °C, 3 s followed by 60 °C for 30 s, for 40 cycles.

To determine the relative amounts of HIV gRNA and β-actin in each intracellular sample, *C*_t_ values were interpreted against standard curves generated on the same qRT-PCR plate under the same conditions, using known quantities of β-actin standard DNA template reagents (401970 TaqMan, Thermo Fisher Scientific) or pSVC21ΔBglII. The β-actin primer sequences and qPCR cycling conditions are described below. β-Actin cDNA template concentrations were in the range of 500 pg–4 ng. Intracellular HIV-1 gRNA levels were then normalized against the β-actin RNA level in each sample versus the average β-actin level found in WT samples, using the following formula: average β-actin level of WT samples/β-actin level of sample. This value was then used to multiply the HIV gRNA content of each sample. The HIV gRNA value of each extracellular sample was then divided by the normalized HIV gRNA level in the corresponding intracellular sample. Outliers were defined as values > the value of the third quartile plus 1.5× the interquartile range, or < the value of the first quartile minus 1.5× the interquartile range [19] 8% of values, evenly distributed between WT, NSC and Δp1 samples, fell into this range and were excluded from further analyses. All data are presented as levels relative to the wild-type average, which was set to 1.

### Western blots

293T cells were transfected and treated with NSC as above. Supernatants were removed and clarified to remove cellular debris at 16,000×*g* for 2 min. Viral particles were inactivated with 0.1% Empigen for 30 min at 56 °C and supernatants were stored at − 80 °C. Cells were washed with PBS and lysed with 500 μL 1× CCLR (cell culture lysis reagent, Promega) for 15 min with gentle rocking, transferred to microfuge tubes and clarified at 13,800×*g* for 1 min before storage at − 80 °C. Samples were denatured at 95 °C for 5 min in a 1:1 (v/v) ratio of Laemmli loading buffer (Biorad) containing 710 mM 2-mercaptoethanol and loaded onto SDS-PAGE gradient gels (Biorad). Gels were electrophoresed at 180 V for 1 h and transferred to nitrocellulose membranes for 20 min at 15 V using Biorad semi-dry transfer apparatus and Towbin transfer buffer (25 mM Tris, 192 mM glycine, 20% (v/v) methanol, pH 8.3). Membranes were blocked in PBST (PBS with 0.05% Tween-20) and 5% (w/v) nonfat milk powder for 30 min at room temperature and incubated with monoclonal anti-Gag p55/p24 (ARP 313, CFAR) in 2.5% (w/v) nonfat milk powder in PBST overnight at 4 °C. Membranes were washed for 4 × 10 min with PBST and incubated with secondary antibody, anti-mouse HRP antibody, (Cell Signalling Technology) for 30–60 min at room temperature, before washing with PBST and PBS and developing with ECL reagent (Thermo Fisher Scientific) according to the manufacturer’s instructions. Bands were quantitated using Image J analysis. Intracellular Gag levels were normalized against GAPDH levels by stripping and reprobing the same blots with anti-GAPDH antibody (Abcam).

### Gag p24 ELISA

96-well microplates (white, half-area, high binding, Greiner Bio-One) were coated overnight with 25 µl of 10 µg/ml anti-HIV-1 p24 Gag antibody (D7320, Aalto Bio Reagents) in 0.1 M NaHCO_3_. The following day, the coating antibody was removed and plates were blocked for 1 h in 5% Bovine Serum Albumin (Sigma-Aldrich) in 1× TBS, and then washed four times in 1× TBS. Plates were loaded with 25 µl of inactivated sample and a standard curve of HIV-1 p24 antigen (AG6054, Aalto Bioreagents), ranging from 0.1 to 10 ng/ml, diluted in 0.05% Empigen-TBS and incubated for 1.5 h. Plates were washed in TBS as above and incubated with anti-HIV-1-p24 alkaline phosphatase conjugate (BC1071-AP) (Aalto Bioreagents) diluted 1:16,000 in 2% milk, 20% sheep serum (Sigma) 0.05% Tween-20 in 1× TBS for 1 h. Plates were washed 4× in PBS-0.1% Tween 20 and incubated with Lumiphos Plus (Lumigen) for 30 min in the dark. Luminescence levels were measured on a GloMax Multi + Multimode Reader instrument (Promega).

### Infectivity assay

To measure infectivity, 6 × 10^4^ TZMbl cells were plated per well in 24-well plates. The following day, 70 µl of virus-containing medium, or medium only, was mixed with 12.5 µl DEAE dextran (50 µg/µl) and added to each well containing TZMbl cells in a total volume of 500 µl DMEM containing 10% (v/v) FCS, 100 U/ml penicillin and 100 μg/ml streptomycin (w/v). 24-h post-transduction, the cells were lysed in 150 µl 1× CCLR buffer (cell culture lysis reagent, Promega) for 15 min at room-temperature. 5 µl of cell lysates was added to a white, half area, medium binding, 96-well microplate (Grainer Bio-One) and 25 µl of Luciferase Assay Reagent (Promega) was added through injectors with a GloMax Multi + Multimode Reader instrument (Promega) and luminescence levels were measured with the following settings; 0.5 s delay, speed 100 µl/s and a 2 s integration time. The average background reading from control cells transduced with medium only was subtracted from the reading from each experimental sample. These background values were less than 2% of the value of the experimental readings. Data were normalized to the average of the wild-type in each independent experiment.

### Integration assay

Jurkat cells were prepared to a density of 5 × 10^5^ cells/well in 24-well plates on day 1 in 500 µl medium. On day 2, Jurkat cells were pre-treated with NSC or raltegravir (NIH AIDS Reagent Program) for 6 h in a final concentration of 100 or 1 µM respectively in triplicate wells. After 6 h, 500 µl pLAI-containing supernatant at a p24 concentration of 365 ng/ml was added to each well, or culture medium was added to control wells. The initial drug concentration was thereby reduced by half during the infection step and the infection was continued for 24 h in a final concentration of 50 µM NSC or 500 nM raltegravir (NIH AIDS Reagent Program). 24 h post- infection, cells were harvested by centrifugation for 5 min at 664×*g* and the DNA was extracted with DNeasy Blood and Tissue kit (Qiagen), according to the manufacturer’s instructions with the following minor modifications; The incubation step with Buffer AL, was performed for 30 min at 56 °C, and the DNA was eluted in nuclease-free water (Thermo Fisher Scientific).

### *Alu*-*Gag* PCR

Integrated HIV DNA was amplified with the *Alu*-*gag* PCR based on a previously published method [20]. 1 μg of extracted DNA was used as template in each PCR containing 1× Colorless GoTaq Reaction buffer (Promega), 100 nM Alu Forward primer: 5′GCCTCCCAAAGTGCTGGGATTACA-3′ and 600 nM *gag* Reverse primer: 5′GTTCCTGCTATGTCACTTCC-3′ [20] PCR nucleotide mix (Promega) [200 µM], Go Taq G2 DNA polymerase (M7841 Promega) 2.5 U, in a final reaction volume of 50 µl. Samples were heated to 95 °C for 2 min. The DNA was amplified during the 40 PCR cycles as follows: 95 °C, 15 s; 50 °C, 15 s; 72 °C, 5 min.

The *Alu*-*gag* PCR products were purified with the QIAquick PCR purification kit (Qiagen) according to the manufacturer’s instructions. The purified PCR products were eluted in 30 µl nuclease free water (Ambion).

### Late RT PCR

Late RT PCR products were amplified by PCR using the pR 5′-AGACCAGATCTGAGCCTGGGAG-3′ and pMA’ 5′-CTGACGCTCTCGCACCC-3′ primers at a final concentration of 100 nM for each primer [21]. 400 ng DNA, extracted from LAI-infected or uninfected control cells, was used as template in each PCR reaction containing 1× Green GoTaq Reaction buffer (Promega), 200 µM PCR nucleotide mix (Promega), 100 nM pR primer, 100 nM pMA’ primer, and Go Taq G2 DNA polymerase (M7841 Promega) at a final concentration of 1.25 U, in a reaction volume of 50 µl. The PCR cycling conditions were as follows; 95 °C, 2 min; 40 cycles at 95 °C, 30 s; 64 °C, 30 s; 73 °C, 30 s; followed by 73 °C for 5 min.

### HIV-1 *gag* PCR

To quantify the amount of integrated HIV-1 DNA, 2 μl of the purified *Alu*-*gag* PCR products were used as template in qPCR in a final volume of 10 µl and subjected to HIV *gag* qPCR as described above with 25 nM HIV *gag* forward primer and HIV 25 nM *gag* reverse primer and 100 nM HIV *gag* probe. 20–40 ng non-*Alu*-*gag* amplified DNA template was included as negative control for background levels.

### *Beta*-*actin* qPCR

20 ng of input DNA for the *Alu*-*gag* PCR was used as template to amplify *β*-*actin* with the following primers: *β*-*actin* forward primer: 5′-GAGCGGTTCCGCTGCCCTGAGGCACTC-3′ and *β*-*actin* reverse primer: 5′-GGGCAGTGATCTCCTTCTGCATCCTG-3′ at 40 nM each (final concentration) [22] in a qPCR reaction containing 2× Fast SYBR Green Master Mix (Applied Biosystems). *β*-*Actin* was amplified with the following qPCR program on a 7500 Fast Real Time PCR System (Applied Biosystems): Step 1. 50 °C for 2 min. Step 2. 95 °C for 20 s. Step 3. 95 °C for 3 s followed by 60 °C for 30 s. Step three was repeated for 40 cycles. A dissociation stage was included at: 95 °C for 15 s, 60 °C for 20 s, 95 °C for 15 s, 60 °C for 15 s.

### In vitro dimerization assays

Nucleotides 1–411 of the genome were prepared by in vitro transcription using DNA templates and purified as described previously [23]. For each sample, 10.5 pmol RNA was renatured by heating to 95 °C for 2 min, snap-cooling on ice for 2 min and diluting to 1 μM in 10 mM Tris–HCl, 140 mM KCl, 1 mM NaCl, 1 mM MgCl_2_. NSC was added at 10× molar ratio either before or immediately after snap-cooling. RNA was incubated at 37 °C for 1–2 h and resolved by native agarose gel electrophoresis alongside an RNA ladder (Riboruler low range, Thermofisher Scientific) and ethidium bromide staining. Bands were visualized on a UV transilluminator and quantified using Image J software. Each independent experiment was performed using a different in vitro transcribed RNA preparation.

### SHAPE analysis of RNA

RNA was transcribed and purified as described [23]. For each sample, 21 pmol of wild-type or Δp1 packaging signal RNA was renatured by heating to 95 °C for 2 min, snap-cooling on ice for 2 min and diluting to 1 μM in 10 mM Tris–HCl, 140 mM KCl, 1 mM NaCl, 1 mM MgCl_2_. RNA was incubated at 37 °C for 2 h and each sample was divided into two equal volumes. One half was treated with a 10× molar ratio of NSC in DMSO for 10 min at room temperature (RT) and the other received an equal volume of DMSO only. Tubes were again divided into two equal volumes before addition of 10 mM (final concentration) NMIA in DMSO or DMSO only. The RNA samples were purified by ethanol precipitation after 45 min incubation at 37 °C and reverse transcribed as described previously. Samples were analyzed by capillary electrophoresis in an ABI 3730 XL sequencer and quantified with SHAPEfinder software [19]. Data were normalized as described previously [24]. Outliers were defined as above and were removed from the dataset. These constituted less than 8% of values. The average reactivities were then used as SHAPE constraints in RNAstructure [25] to predict structural models and their free energies.

## Results

### Early phases of the viral lifecycle from entry through integration are unaffected by NSC

**Fig. 1:**
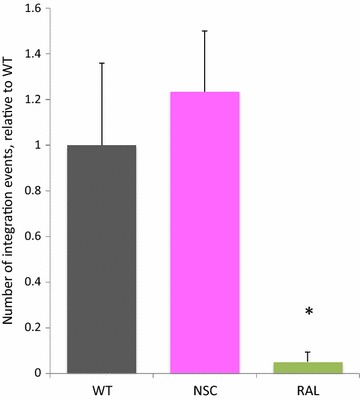
Viral integration levels determined in the presence and absence of NSC and integration inhibitor. Jurkat cells were pre-treated with NSC [100 µM] or raltegravir [1 µM], and infected with LAI 6 h post-treatment in the presence or absence of 50 µM NSC or 500 nM raltegravir. 24 h post-infection, cells were harvested and subjected to DNA extraction and HIV-1 integration was assayed by *Alu*-*gag* PCR followed by quantification of integration events by HIV-1 *gag* qPCR. The number of integration events relative to LAI-infected cells (WT) in the presence of NSC or raltegravir is shown. For each sample, variations in the amount and quality of cellular DNA input was accounted for by dividing the number of HIV-1 integration events detected by the HIV-1 *gag* qPCR by the relative actin level detected in the input DNA for the *Alu*-*gag* PCR. All samples were then normalized relative to one another such that the average WT value was 1. The average of three samples is shown for each condition. Error bars represent SD. *Statistically significant from WT by Student’s *t* test, *p* < 0.05)

NSC was previously observed to prevent Gag interacting with SL3 in vitro and to stabilize the RNA. Structural rearrangement of the RNA, as well as its interaction with viral proteins are integral events during reverse transcription [26–28] and potentially vulnerable to an intervention that prevents protein interaction and stabilizes SL3 structure. Conceivably a nucleotide binding agent might also interfere with the integration step. Perturbation of either of these processes would be detectable by infecting NSC treated/untreated cells with wild-type virions and quantitating integration frequency. Jurkat cells were thus pre-treated for 6 h with 100 μM NSC in 500 µl medium or untreated, and then mixed with an equal volume of medium containing HIV LAI. Viral integration events were determined by *Alu*-*gag* PCR and HIV-1 *gag* qPCR, based on a previously described method to assay for HIV-1 integration [20]. No statistically significant difference in integration events was seen in Jurkat cells in the presence of NSC compared to untreated pLAI-infected cells 24 h post-infection (Fig. 1). pLAI-infected cells treated with the integrase inhibitor raltegravir were included as a positive control for inhibition of integration, and this led to a greater than 95% reduction in integration events (*p* < 0.05 by *t* test). HIV-1 integration is thus unaffected by NSC. A similar trend was observed in two further independent experiments. As NSC is not affecting the levels of integrated provirus seen when cells are pre-treated with the compound by implication it is unlikely that the compound interferes with the reverse transcription process, since this should also lead to a decrease in the number of integration events. We confirmed this by briefly investigating reverse transcription by PCR on extracted DNA using the primers pR and pMA’ as previously described [21] to detect late reverse transcription products and did not see a change in reverse transcription upon treatment with NSC (data not shown).

### Viral Gag production and budding are not significantly affected by NSC treatment

**Fig. 2:**
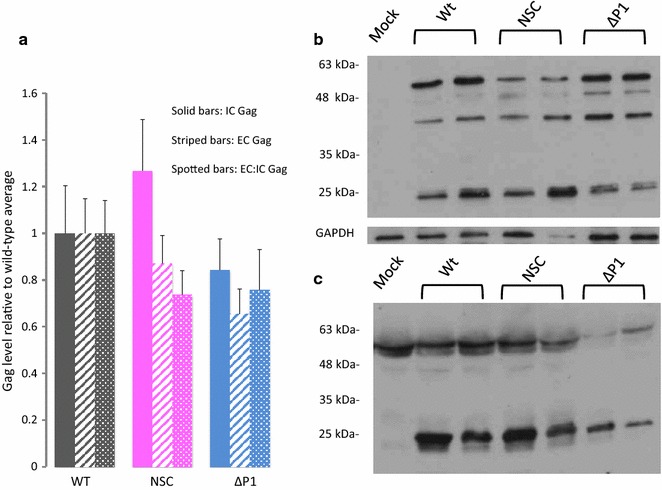
Viral Gag production in the presence and absence of NSC. 293T cells were transfected with HIVΔEnv (WT and NSC) or HIVΔp1ΔEnv (Δp1) and VSV-G expression plasmids. 6 h post-transfection, cells were treated with 50 μM NSC (NSC) or the equivalent quantity of DMSO (WT and Δp1). 24 h post treatment, supernatants and cells were harvested. Equal volumes of samples were electrophoresed on SDS-PAGE gels and immunoblots performed using anti-p24/p55 antibody. Cellular blots were stripped and reprobed with anti-GAPDH antibody. **a** Results of cellular, supernatant and supernatant:cellular Gag expression levels, quantified using densitometry. Results show an average of four independent experiments, each containing 3–6 replicates (with each replicate examining an independently transfected well of a six-well plate) of each experimental condition. **b** Anti-Gag Western blot showing intracellular Gag expression levels, and anti-GAPDH control. Two experimental replicates, from independently transfected wells are shown (**c**) anti-Gag Western blot showing extracellular Gag expression. Two experimental replicates from independently transfected wells are shown

In the previous study ELISA assays on levels of supernatant p24 suggested the existence of lowered virion release from the cell in the presence of NSC. We reinvestigated this finding as the previous study did not take into account the intracellular p24 levels in each sample. Initial ELISA experiments, performed as previously, corroborated an apparent reduction in particle release as they showed around a 40% level of p24 in virions purified from equal volumes of supernatant in the presence of NSC versus wild-type. Strikingly this level was similar to that seen with the Δp1 virus; both results being statistically significant to *p* < 0.05 (Additional file 1). However, when intracellular p24 production was accounted for (Additional file 1: IC and EC:IC bars) the data were no longer statistically significant. As discussed below detection of p24 levels depends on effective Gag processing and HIV RNA structure can affect the efficiency of viral protease cleavage of the Gag protein during particle maturation [10, 29]. Accordingly, we also assessed the production of Gag protein inside and its export outside cells by Western blot in addition to ELISA to ensure that we were detecting all Gag protein species regardless of their conformation. Using identical preparatory conditions to our qRT-PCR samples, the total amount of Gag observed in cellular fractions was measured by densitometry and normalized to GAPDH levels (Fig. 2a solid colour bars and Fig. 2b). The amount of Gag present in equal volumes of supernatant (Fig. 2a striped bars and Fig. 2c) was also measured, and the ratio of extracellular to intracellular Gag compared (Fig. 2a, spotted bars). Figure 2a shows an average of four separate experiments each containing 3–4 independent transfections of each of wild-type, NSC-treated and Δp1 extracts. No statistically significant difference from wild-type was detected when assessed in any of these independent experiments by *t* test and, crucially, no trend was apparent either. As levels of Gag protein were variable between samples of all three types, as can be seen in Fig. 2b, c, we compiled data from all four independent experiments to ascertain whether a difference would be observed with a larger sample size. Again, no significant difference was detected when data from all four experiments were compared, as presented in Fig. 2a, although on average the relative quantity of viral particles in the supernatant after NSC treatment was 0.87 of that of wild-type, and 0.65 for SL3 deletion virus. This suggests that there may be a small defect in particle release upon NSC treatment, and a larger defect upon SL3 deletion, but that neither of these is statistically significant using sample sizes of up to 13 replicates. Although solution based methods of quantitation are often thought to be more robust, the main difference in detection mechanism between ELISA and Western blot assays is that Western blots analyze all Gag products, regardless of their 3D conformation whereas the ELISA assay measures the major Capsid protein, p24, in a specific 3D structure. The ELISA assay may thus be influenced by structural perturbations in the Gag protein, in a way that the Western blot assay is not and, in these circumstances, Western blots provide more accurate data on the level of Gag protein released from cells.

### In the presence of NSC, the level of viral RNA in the cytoplasm is unaffected but levels released into the supernatant are reduced

**Fig. 3:**
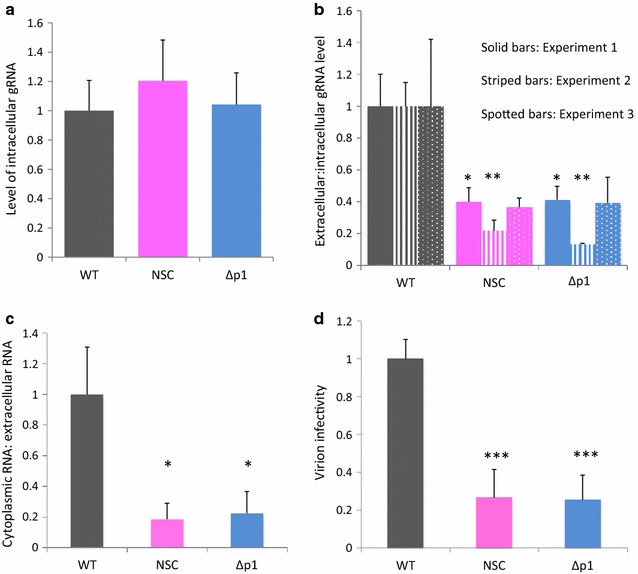
Viral gRNA levels analyzed by qRT-PCR, in the presence and absence of NSC. 293T cells were treated as in Fig. 2. 24 h post treatment, supernatants and cells were harvested, virions purified from 500 μL supernatant and RNA extracted. **a** gRNA levels in cells, analyzed by qRT-PCR. Each sample was first normalized against β-actin levels and then normalized against the average wild-type gRNA levels. Data shown are the average of 14 –18 samples taken from four independent experiments. Error bars represent SD. **b** Ratio of gRNA levels in purified virions from equivalent volumes of supernatant to gRNA levels inside cells. Intracellular levels were first normalized against β-actin; these values were then used to divide the virion RNA levels for each sample. Data were then normalized against the wild-type average. Data are shown as three independent experiments, each containing 4–9 replicates. **c** Ratio of gRNA levels in purified virions from equivalent volumes of supernatant to cytoplasmic gRNA. Data are representative of two independent experiments. **d** Ability of virions produced in the presence or absence of NSC to transduce cells, measured by luciferase assay. Error bars represent the SD. *p < 0.05; **p < 0.01 by Student’s *t* test. Data are representative of two independent experiments

The interaction of Gag with SL3 has been proposed by many groups to be important in the packaging process [13, 30–32]. In the absence of NSC, Gag protein was shown to interact with an isolated SL3 RNA stem loop in vitro causing it to unwind and unmasking a fluorophore attached to the 5′ end from a quencher on the 3′ end of the RNA stem-loop. The presence of NSC prevented this unwinding [7, 9]. In a single round infectivity experiment it led to reduced levels of infectious virion production as determined by a Tat-sensitive reporter cell-based assay [9]. In order to ascertain whether, by preventing the native interaction of Gag with SL3, NSC is able to specifically lower gRNA levels inside viral particles, we measured gRNA levels in cellular lysates and purified virions by qRT-PCR in the presence or absence of NSC, with the Δp1 virus serving as a positive control for inhibition of packaging [13]. Samples transfected with pBluescript alone were included as a negative control in each experiment and these did not generate a detectable signal by qRT-PCR, as expected (data not shown). The level of gRNA found in cells was unaffected by the presence of NSC or by deletion of SL3, as shown in Fig. 3a, with no statistically significant differences from wild-type observed by *t* test. However, when the ratio of gRNA incorporated into virions was compared to intracellular levels a clear reduction was observed in the presence of NSC to a similar level to that seen in the SL3 deletion virus: Genomic RNA was reduced to approximately 35% of wild type levels in the presence of NSC compared to 34% for the SL3 deletion virus (Fig. 3b). Δp1 results were very similar to those observed previously by RPA assay [14]. It is possible that these results could reflect a difference in nuclear RNA metabolism or export, as a quinolinium derivative such as NSC could conceivably diffuse into the nucleus and affect these cellular functions. To ascertain whether the decrease in virion gRNA level upon NSC treatment we observed was specific to the packaging process we performed a nuclear:cytoplasmic fractionation and measured cytoplasmic gRNA levels against virion gRNA levels by qRT-PCR as before. The ratio of virion:cytoplasmic gRNA was 18.5% upon NSC treatment and similar to that of the Δp1 virus (22.3%) (Fig. 3c), suggesting that the compound acts after the cytoplasmic phase of the viral lifecycle.

The greater than five fold reduced level of gRNA in the presence of NSC compared to the wild type virus clearly cannot be accounted for by the possible minor reduction in export of Gag. The similarity to the phenotype of an established packaging defective mutant supports that NSC is primarily an inhibitor of RNA encapsidation. Taken together, these results suggest that NSC is specifically lowering the amount of viral RNA inside viral particles.

To further confirm this we measured the infectivity of virions produced in the presence of NSC. The reporter cell line TZMbl was inoculated with equivalent volumes of virus-containing medium and the infectivity measured compared to WT and Δp1 virus 24 h post-infection by luciferase assay. The reduction in infectivity in the presence of NSC was of a similar magnitude to the reduction in gRNA levels detected in virions in the supernatant (26% of wt, Fig. 3d). The effect of the drug was to decrease viral infectivity to a similar level to that seen in the Δp1 virus.

### NSC does not prevent gRNA dimerization, but does have widespread stabilizing effects on the 5′UTR and packaging signal RNA structure

**Fig. 4:**
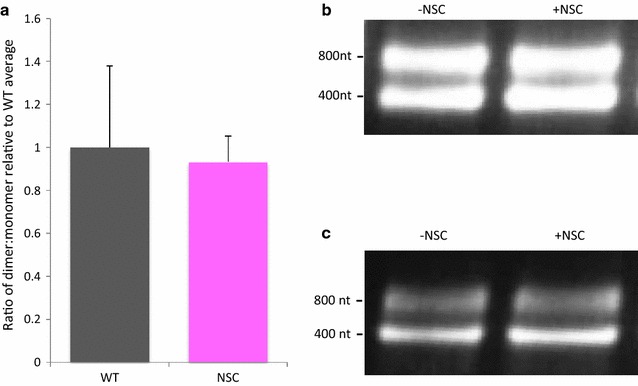
Effects of NSC on dimerization of viral RNA. In vitro dimerization assays in the presence and absence of 10× molar ratio of NSC. RNA was heated to 95 °C and NSC in DMSO, or DMSO only, was added before snap-cooling on ice. RNA was then incubated for 1–2 h before native gel electrophoresis and densitometry analysis. **a** Dimerisation levels relative to the untreated RNA. Data are an average of five independent experiments, containing 1–3 samples of untreated/treated RNA each. Error bars show SD. **b** Representative samples from RNA renatured in the absence or presence of NSC, with (**b**) 2 h incubation at 37 °C or (**c**) 1 h incubation at 37 °C

gRNA dimerization and RNA encapsidation are intimately linked and interdependent. Although NSC interacts with the loop region of SL3, and SL3 has not been implicated as an important component of the dimerization process, it is possible that NSC is causing inhibition of packaging as a secondary effect by interfering with the ability of the viral gRNA to dimerize. In order to ascertain whether NSC acts by preventing dimerization we performed in vitro dimerization assays in the presence and absence of NSC. Packaging signal RNA (nts 1–411 of the genome) was in vitro transcribed and refolded under dimerization inducing conditions as previously described [23, 33] and examined by native agarose electrophoresis and densitometry. NSC was added either before RNA snap-cooling and refolding (data shown in Fig. 4a, b) or after snap-cooling and before refolding (data not shown). During the in vitro dimerization process, the formation of dimer is a dynamic process, with the amount of dimer increasing over time. Thus we also examined dimerization at an earlier timepoint, (Fig. 4c) to verify that the levels of dimer that form are changing with time in a similar manner, with or without NSC treatment. No significant differences were observed, suggesting that NSC is not blocking the ability of the viral RNA to form dimers efficiently.

**Fig. 5:**
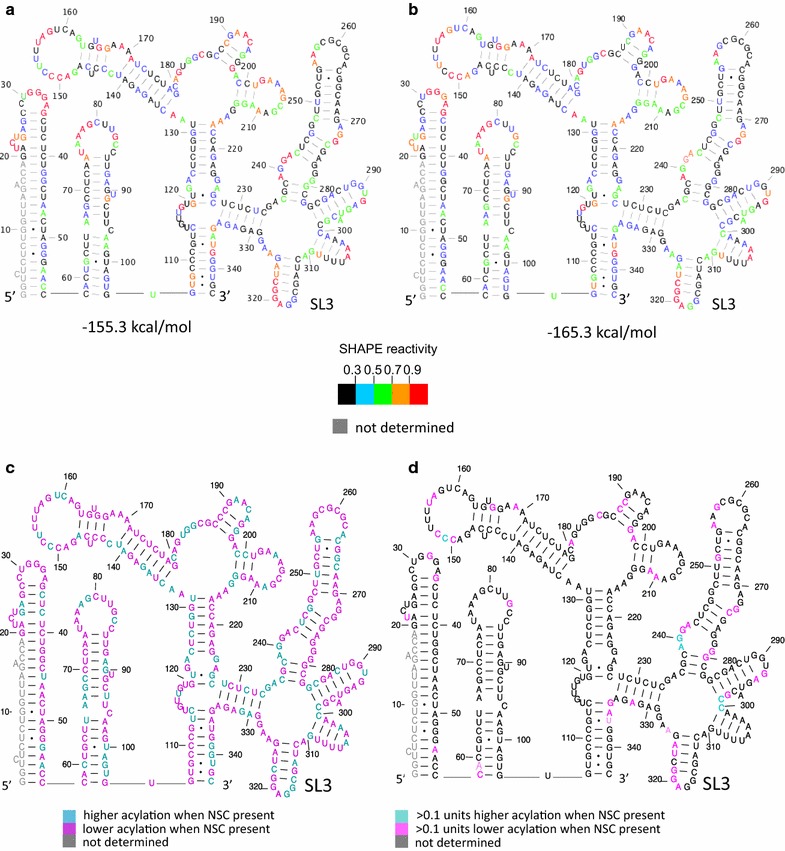
Structural effects of NSC on the gRNA. The first 411 nts of the gRNA, containing the packaging signal region and beginning of the *gag* ORF were in vitro transcribed and refolded. RNA was treated with NSC in DMSO or DMSO only. Acylation sensitivity at each nucleotide was used to model the structure and predicted free energy. The most stable structure is shown. **a** Predicted RNA structure and stability of DMSO treated RNA. **b** Predicted RNA structure and stability of NSC treated RNA. **c**, **d** Acylation sensitivity difference upon NSC treatment. **c** Differences > or < 0 and **d** differences > or < 0.1 reactivity units

NSC was originally identified as a potential antiviral by it inhibiting the ability of Gag to unwind a synthetic SL3 RNA molecule. However it was unclear whether the major mechanism of action of NSC was prevention of Gag binding to SL3, or from prevention of the unwinding of SL3 although there was evidence of the latter effect from a rise in melting temperature of SL3 when NSC was bound [9]. To further investigate the structural effects of NSC on RNA structure of the viral 5′UTR we performed SHAPE (selective 2′OH acylation analyzed by primer extension) analysis of the dimeric RNA in the presence and absence of the compound. SHAPE compounds react with the RNA backbone where it is flexible, which occurs mainly in single-stranded regions, and are used to inform structural modeling. In vitro transcribed RNAs were renatured under conditions that favor dimerization, as before. RNAs were treated with NSC in DMSO or DMSO only before probing with NMIA (N-methyl isatoic anhydride) and fragment analysis as performed in previous studies [23, 24]. 12 renatured samples were divided into two to be probed with/without NSC and divided into two again to be probed with/without NMIA, using 6 different in vitro transcription preparations of RNA. Data were analyzed with SHAPEfinder software. Structures were modeled using RNAstructure [25] and are shown in Fig. 5a–d, with numerical data in the Additional file 2: Table S1. Backbone acylation sensitivity at each nucleotide is shown in Fig. 5a for wild-type RNA without NSC and for wild-type RNA with NSC in Fig. 5b. These data show that the action of NSC on the packaging signal RNA structure is to stabilize the sugar–phosphate backbone structure, reducing its conformational flexibility. This is best illustrated by the free energy change upon NSC treatment (− 155.3 to − 165.3 kcal/mole). Although most acylation differences upon NSC treatment are small, the vast majority of nucleotides are less reactive to NMIA after NSC treatment, and this extends across the RNA, as is shown in Fig. 5c, which illustrates the reactivity differences before and after NSC treatment. This stabilization occurs at SL3 in particular, as shown in Fig. 5d by the larger reduction in SHAPE reagent acylation of five of the SL3 nucleotides, but additional effects can be seen extending across the entire psi region. To ascertain whether these results were due to off-target, nonspecific effects of NSC binding to other regions of the RNA, we repeated the SHAPE experiments using the Δp1 RNA, with and without NSC treatment. This RNA has a 19 nt deletion at SL3, but is otherwise isogenic. In contrast to the wild type RNA, NSC did not stabilize this transcript (Additional file 3: Fig. S2; Additional file 2: Table S1). This implies that the stabilization of SL3 is responsible for a propagated increase in structural rigidity rather than NSC being a non-specific RNA stabilizing agent.

## Discussion

We investigated the ability of NSC to block HIV-1 replication, focusing on individual life cycle stages. When virus is produced in the presence of NSC, this RNA-binding compound specifically reduces the amount of gRNA in virions in the supernatant fraction, compared to the amount of gRNA available for packaging in the cells. This is possibly associated with a non-significant minor decrease in the amount of Gag protein detected in the supernatant in the presence of NSC. The dominant mechanism of action of NSC is to specifically prevent the incorporation of viral gRNA into virus particles with a comparable defect to that produced in an established packaging defective viral mutant, the Δp1 virus. The infectivity of the virions produced upon NSC treatment correlated well with the reduction in gRNA packaging. No effects were seen on other stages of the viral lifecycle again confirming that NSC has a specific mechanism of action at the packaging stage of the viral lifecycle.

We cannot fully exclude that equal amounts of gRNA are initially incorporated into virions but that NSC renders the particles less stable, and they disintegrate faster. Although possible, we think this explanation is less plausible, as a previous study using a SL3 deletion virus showed no reduction of p24 protein in the virion- associated fraction, versus wild-type virus [34]. It is also possible that NSC does not prevent the initial stages of packaging, but instead destabilizes the RNA inside virions, leading to degradation of the RNA. In practical terms the effect is the same and gRNA levels in virions are lowered, which would be an equally desirable therapeutic outcome.

**Fig. 6:**
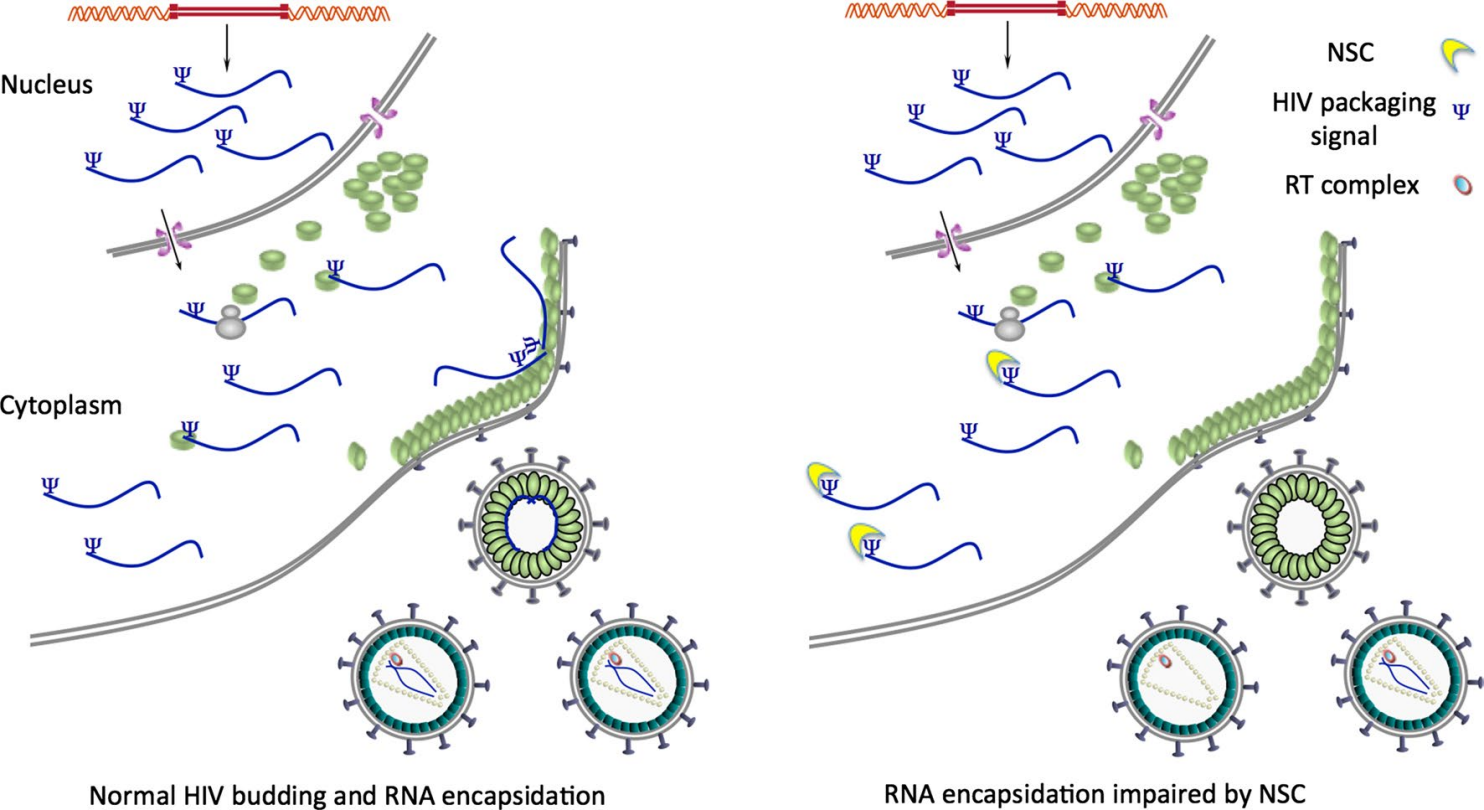
Schematic diagram showing the RNA packaging impairment upon NSC treatment. NSC may also be present inside virions

Our findings are summarized schematically in Fig. 6.

We observed that ELISA and Western blot can give differing results for Gag protein levels in the presence of RNA binding agents or RNA structural deletion mutants. These findings resonate with the known effects of RNA on structural stability of HIV virions [35] and may possibly relate to effects of RNA on Gag processing [36]. This observation has implications for investigation of viral processes that involve different structural changes in Gag and suggests that results should be validated by Western blots, or by alternative analyses relying on quantification of denatured proteins rather than relying only on methods detecting the native state. This is particularly pertinent as the viral maturation process involves multiple cleavages and structural rearrangements of the Gag protein, such as the formation of a new beta-hairpin structure in CA, necessary for proper core formation [37, 38]. Although large and small-scale structural rearrangements of the Gag polyprotein have been proposed to occur [38, 39] little is known about the structures of the cleavage intermediates. In addition, incorrect cleavage order has been shown to affect the viability of virions [40, 41].

HIV-1 RNA has been suggested to adopt a range of different conformers, with each one playing a different role in the viral lifecycle [10, 42, 43]. The SHAPE data presented here illustrate that NSC not only enhances the stability of SL3 but that this also stabilizes the entire packaging signal region, reducing its conformational flexibility. This result suggests that by stabilizing SL3, the ‘BMH’ form [44] of the RNA is stabilized. In this way, a structural stabilization at one site propagates across the structure, having wide-reaching effects. Acylation sensitivity differences within single-stranded regions are likely to be due to stabilization of the backbone into more or less reactive noncanonically paired conformations, such as by differences in sugar-pucker or noncanonical interactions [45]. Recent publications indicated that although TAR-binding compounds had small longer-range structural effects outside TAR [46, 47], they did not have such far-reaching stabilizing effects. The structure of SL3 is usually similar between structural models of the HIV-1 leader region produced by different groups [48–50] and it has not been proposed to be an integral part of conformational switches in the viral RNA. However, the stabilization of a BMH-like structure by NSC binding to SL3 suggests that unwinding or stabilization of the SL3 site may play a pivotal role in controlling structural changes within the HIV-1 leader RNA.

SL3 has recently been proposed to be of lower importance for Gag binding and viral packaging than nearby motifs [51], however, we show here that a compound that specifically binds to it has reasonably large effects on viral packaging efficiency, making a case for its importance in the packaging process.

Interestingly, our findings indicate that an HIV-1 RNA which is stabilized at SL3 does not show defects at other stages of the lifecycle, suggesting that structural flexibility imparted by this region is not necessary for other lifecycle stages.

Although our results show that in vitro, NSC does not influence the ability of the viral RNA to form dimers, we cannot exclude that it affects the structure or the dynamic stability of the dimers that do form in vivo. A recent model suggests that it is not simply SL1 that remodels into an intermolecular duplex, but also extensive regions around SL1, including the U5:AUG interaction, which was previously thought to be intramolecular [52]. SL3 is positioned in the middle of this proposed intermolecular interaction and its remodeling may be important for formation of the extended intermolecular complex.

## Conclusions

By binding to the SL3 RNA in ψ, NSC specifically inhibits the incorporation of HIV gRNA into virions. It does this without affecting earlier processes of the viral lifecycle such as reverse transcription, integration, production and nuclear export of viral gRNA or the steady-state intracellular Gag protein levels. NSC260594 is thus a first-in-class RNA-binding anti-packaging compound. As a small molecule that affects RNA structure and function, it can be used to interrogate the role of RNA structure in the viral lifecycle without the need for mutation of the viral genome.

## Additional files

**Additional file 1: Figure S1.** p24 concentration measured by ELISA. 293T cells were transfected and treated as for Fig. 2. Error bars represent standard deviation. Data shown is for three independent experiments *p < 0.05.

**Additional file 2: Table S1.** Table: Numerical representation of SHAPE reactivity differences at each nucleotide position. Colours shown are the same as those in Fig. 5 and Additional file 3: Fig. S2.

**Additional file 3: Figure S2.** Structural effects of NSC on the Δp1 RNA. Δp1 RNA was in vitro transcribed, refolded and treated with DMSO only or NSC in DMSO as for Fig. 5. Acylation sensitivity at each nucleotide was used to model the structure and predicted free energy. The most stable structure is shown. **a** Predicted RNA structure and stability of DMSO treated RNA. **b** Predicted RNA structure and stability of NSC treated RNA.
