# Supplementary figures and images for: An RNA-binding compound that stabilizes the HIV-1 gRNA packaging signal structure and specifically blocks HIV-1 RNA encapsidation
Source: Retrovirology. 2018 Mar 14;15:25. doi: 10.1186/s12977-018-0407-4 (PMC5853050; doi:10.1186/s12977-018-0407-4)

## Slide 1
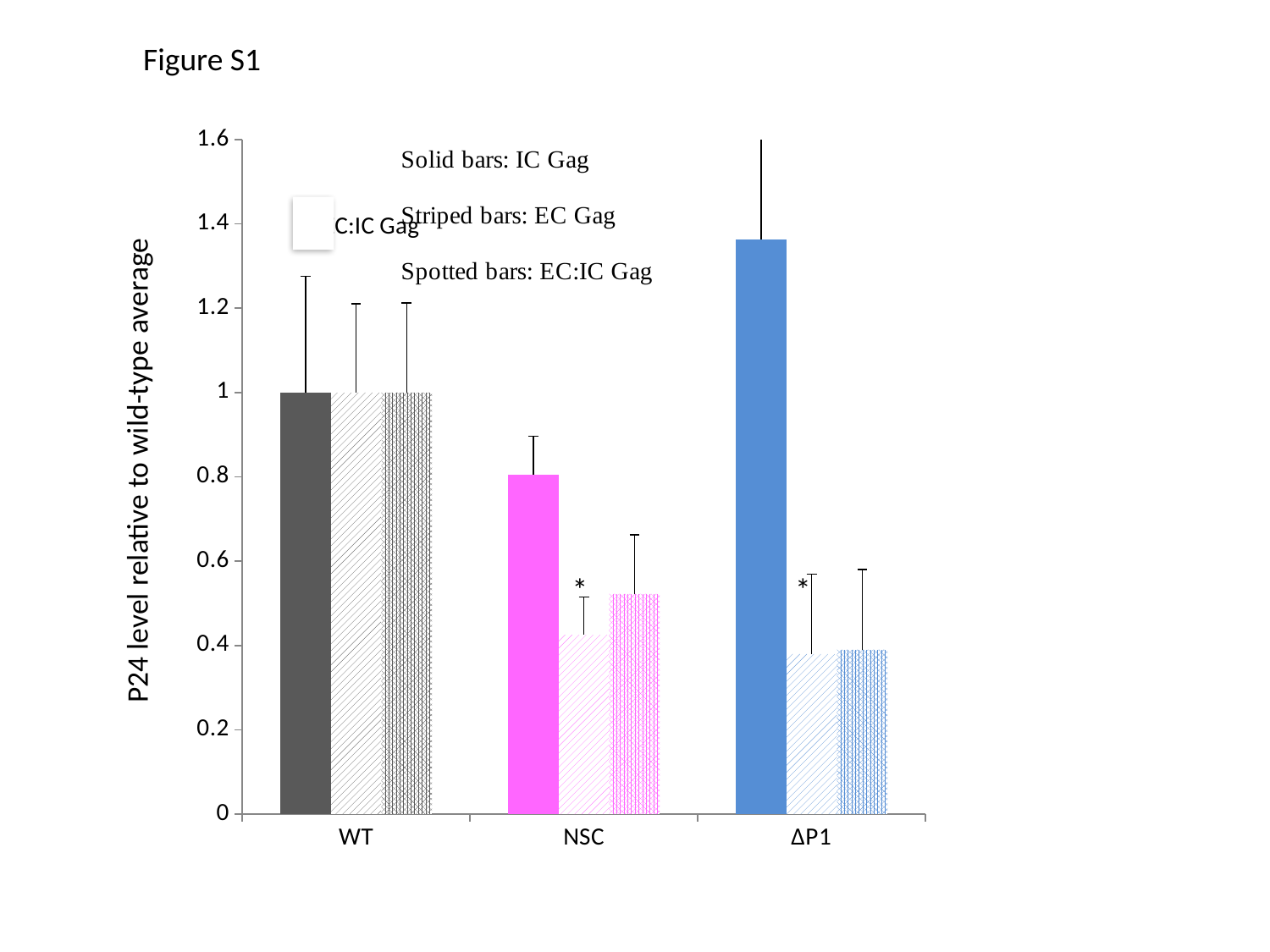

Figure S1
### Chart
| Category | | | |
|---|---|---|---|
| WT | 1.0000000292852234 | 0.99984155022023 | 0.999999993764844 |
| NSC | 0.805023519652397 | 0.42538866842117506 | 0.522384977152093 |
| ΔP1 | 1.3633771637661583 | 0.37969720104881305 | 0.38986620129390814 |
P24 level relative to wild-type average
*
*

Supplement: Supplementary file 1 — Additional file 1: Figure S1. p24 concentration measured by ELISA. 293T cells were transfected and treated as for Fig. 2. Error bars represent standard deviation. Data shown is for three independent experiments *p < 0.05. [file 12977_2018_407_MOESM1_ESM.pptx]

## Slide 1
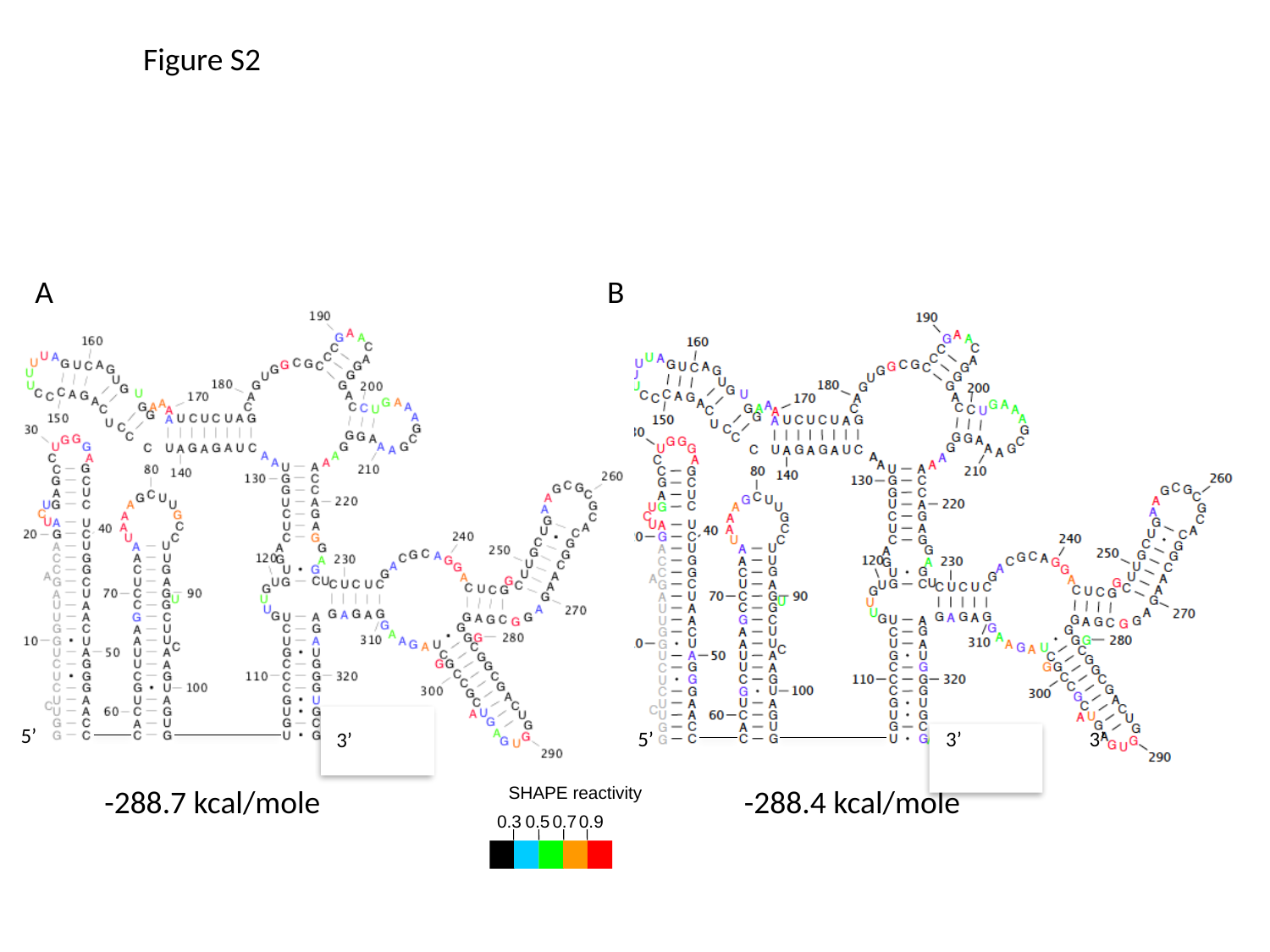

Figure S2
A
B
5’
5’
3’
3’
3’
-288.7 kcal/mole
SHAPE reactivity
0.3
0.5
0.7
0.9
-288.4 kcal/mole

Supplement: Supplementary file 3 — Additional file 3: Figure S2. Structural effects of NSC on the Δp1 RNA. Δp1 RNA was in vitro transcribed, refolded and treated with DMSO only or NSC in DMSO as for Fig. 5. Acylation sensitivity at each nucleotide was used to model the structure and predicted free energy. The most stable structure is shown. a Predicted RNA structure and stability of DMSO treated RNA. b Predicted RNA structure and stability of NSC treated RNA. [file 12977_2018_407_MOESM3_ESM.pptx]
