# Supplementary material for: An RNA-binding compound that stabilizes the HIV-1 gRNA packaging signal structure and specifically blocks HIV-1 RNA encapsidation
Source: Retrovirology. 2018 Mar 14;15:25. doi: 10.1186/s12977-018-0407-4 (PMC5853050; doi:10.1186/s12977-018-0407-4)
Supplement: Supplementary file 2 — Additional file 2: Table S1. Table: Numerical representation of SHAPE reactivity differences at each nucleotide position. Colours shown are the same as those in Fig. 5 and Additional file 3: Fig. S2. [file 12977_2018_407_MOESM2_ESM.pdf]

nt number	WT RNA	WT RNA PLUS NSC	DIFFERENCE UPON ADDITION OF NSC
1			
2			
3			
4			
5			
6			
7			
8			
9			
10			
11			
12			
13			
14			
15			
16			
17			
18			
19			
20	0.043498865	0.032466468	-0.0110324
21	0.188494861	0.157422164	-0.0310727
22	0.903669812	0.794064403	-0.10960541
23	0.990048865	0.893584792	-0.09646407
24	0.769654879	0.684376108	-0.08527877
25	0.750201192	0.7539681	0.003766908
26	0.338893376	0.381613173	0.042719797
27	0.443615859	0.427121941	-0.01649392
28	0.265713195	0.264378975	-0.00133422
29	0.093805355	0.058592935	-0.03521242
30	0.531999291	0.44411665	-0.08788264
31	1.262919185	1.235107824	-0.02781136
32	1.990491063	1.770935808	-0.21955525
33	0.907250155	0.862612637	-0.04463752
34	1.06922438	0.994927204	-0.07429718
35	1.650342782	1.455134728	-0.19520805
36	0.282762287	0.307463598	0.024701311
37	0.106108195	0.086616375	-0.01949182
38	0.169549122	0.174062353	0.004513231
39	0.064742568	-0.002123188	-0.06686576
40	0.160896668	0.094457748	-0.06643892
41	0.113376179	0.06696318	-0.046413
42	0.43075352	0.346638182	-0.08411534
43	0.313757198	0.257891122	-0.05586608
44	0.289707959	0.265172507	-0.02453545
45	-0.004507997	0.003328779	0.007836776
46	0.419931666	0.382088878	-0.03784279
47	0.58107028	0.57867868	-0.0023916
48	0.226338446	0.182598449	-0.04374

49	0.112984654	0.084717863	-0.02826679
50	0.250706592	0.227449176	-0.02325742
51	0.387447641	0.310150825	-0.07729682
52	0.425405669	0.335745048	-0.08966062
53	0.16340624	0.197810124	0.034403884
54	0.603430299	0.390236393	-0.21319391
55	0.395551288	0.435794664	0.040243376
56	0.661270559	0.644997176	-0.01627338
57	0.078755529	0.061005977	-0.01774955
58	0.265187566	0.164857819	-0.10032975
59	0.434308548	0.333159219	-0.10114933
60	0.159044324	0.171825898	0.012781574
61	0.329162294	0.281690043	-0.04747225
62	0.547564496	0.516788963	-0.03077553
63	0.245889284	0.239919365	-0.00596992
64	0.09268468	0.084262025	-0.00842265
65	0.265203755	0.293619137	0.028415381
66	0.502447099	0.522355199	0.019908101
67	0.373993647	0.465786015	0.091792368
68	0.476964951	0.533632082	0.056667131
69	0.190824938	0.223738319	0.032913381
70	0.175076245	0.099285826	-0.07579042
71	0.07396834	0.038273199	-0.03569514
72	0.150007435	0.13798085	-0.01202659
73	0.215277848	0.183124611	-0.03215324
74	0.740132158	0.722578026	-0.01755413
75	0.963754115	0.905810876	-0.05794324
76	0.613415951	0.600251769	-0.01316418
77	1.193761582	1.170213991	-0.02354759
78	1.231192305	1.278131846	0.046939541
79	0.975685397	0.981864206	0.006178809
80	0.319335802	0.318815015	-0.00052079
81	0.061571318	0.04153467	-0.02003665
82	0.453633557	0.443447683	-0.01018587
83	0.914617508	0.759638005	-0.1549795
84	0.597357137	0.636116747	0.03875961
85	0.136919492	0.120428289	-0.0164912
86	0.232793308	0.201334805	-0.0314585
87	0.135518516	0.135403034	-0.00011548
88	0.409024108	0.396531869	-0.01249224
89	0.439207675	0.462705961	0.023498286
90	0.176354001	0.210343254	0.033989253
91	0.34609797	0.313702444	-0.03239553
92	0.759379003	0.710690392	-0.04868861
93	0.168221834	0.153590538	-0.0146313
94	0.119597497	0.130817846	0.011220349
95	0.215509762	0.212806216	-0.00270355
96	0.256130839	0.235357889	-0.02077295
97	0.683468441	0.5981483	-0.08532014

98	0.660280805	0.60636645		-0.05391436
99	0.121494355	0.117987907		-0.00350645
100	0.241835932	0.246690888		0.004854956
101	0.285435372	0.313201043		0.02776567
102	0.414899614	0.43332263		0.018423016
103	0.359690232	0.36140062		0.001710388
104	0.279820928	0.238824464		-0.04099646
105	0.648326106	0.59706973		-0.05125638
106	0.413341753	0.399926994		-0.01341476
107	0.753594187	0.743746707		-0.00984748
108	0.781822506	0.692940469		-0.08888204
109	0.216528271	0.199895577		-0.01663269
110	0.213134375	0.117573485		-0.09556089
111	0.091876528	0.063718609		-0.02815792
112	0.251395275	0.160363979		-0.0910313
113	0.219933943	0.232435978		0.012502036
114	0.311759608	0.320491445		0.008731837
115	0.232022244	0.186783419		-0.04523882
116	0.244321532	0.212633657		-0.03168788
117	0.250256878	0.176550131		-0.07370675
118	0.397227295	0.402986102		0.005758808
119	1.056497077	1.02640288		-0.0300942
120	0.19238057	0.162230741		-0.03014983
121	0.755887922	0.747984872		-0.00790305
122	0.043883525	0.072686663		0.028803137
123	0.55792659	0.541346633		-0.01657996
124	0.408183677	0.415815543		0.007631866
125	0.367266766	0.405531707		0.038264941
126	0.176134751	0.162620379		-0.01351437
127	0.047459059	0.056818461		0.009359402
128	0.266147154	0.198540257		-0.0676069
129	0.216094855	0.22755014		0.011455284
130	0.408027544	0.34992556		-0.05810198
131	0.017953648	0.026298799		0.008345151
132	0.575788104	0.556399194		-0.01938891
133	0.932745028	0.933205256		0.000460228
134	0.485657141	0.486681795		0.001024653
135	0.208923049	0.192895693		-0.01602736
136	0.257032552	0.26635225		0.009319698
137	0.387700608	0.422260601		0.034559992
138	0.45077136	0.369396646		-0.08137471
139	0.046485529	0.044565698		-0.00191983
140	0.26303988	0.287981325		0.024941445
141	0.327685936	0.351551902		0.023865966
142	0.552044306	0.614693009		0.062648703
143	0.324667065	0.260790807		-0.06387626
144	0.242299831	0.184738915		-0.05756092
145	0.028232574	0.015977323		-0.01225525
146	0.314202094	0.286247831		-0.02795426

147	0.18167867	0.201592636		0.019913966
148	0.513554034	0.452840312		-0.06071372
149	0.947850792	0.853577569		-0.09427322
150	2.814488221	2.146073601		-0.66841462
151	1.33662144	1.465885281		0.12926384
152	0.471976178	0.256209156		-0.21576702
153	0.685398711	0.71385501		0.028456299
154	0.401778057	0.345956187		-0.05582187
155	0.140483046	0.129133889		-0.01134916
156	1.141916585	1.028620332		-0.11329625
157	1.512981721	1.349758404		-0.16322332
158	0.928508887	0.878367875		-0.05014101
159	0.290148625	0.339279208		0.049130583
160	0.292072662	0.367569816		0.075497154
161	0.185018023	0.155174949		-0.02984307
162	0.594873288	0.567203931		-0.02766936
163	0.501752516	0.453190645		-0.04856187
164	0.078210476	0.050235075		-0.0279754
165	0.442523675	0.391110545		-0.05141313
166	1.19494314	1.075000136		-0.119943
167	0.785385128	0.736384172		-0.04900096
168	0.23002879	0.190297284		-0.03973151
169	1.092314817	0.940790781		-0.15152404
170	0.082275785	0.064948978		-0.01732681
171	1.099097095	1.006402453		-0.09269464
172	0.465837605	0.45897774		-0.00685986
173	0.426271488	0.421155649		-0.00511584
174	0.078651732	0.025459599		-0.05319213
175	0.152278921	0.13017111		-0.02210781
176	0.216045888	0.157415881		-0.05863001
177	0.421920671	0.345333325		-0.07658735
178	0.315009523	0.30134742		-0.0136621
179	0.393469009	0.475620099		0.08215109
180	0.804002899	0.626930192		-0.17707271
181	1.146811264	1.067185182		-0.07962608
182	0.283908283	0.323984607		0.040076324
183	0.525855491	0.556276926		0.030421435
184	0.425497617	0.493744971		0.068247354
185	1.1030333	0.92795156		-0.17508174
186	0.137472035	0.08455051		-0.05292153
187	0.349956083	0.271753844		-0.07820224
188	0.299309049	0.169240799		-0.13006825
189	0.890447404	0.682811625		-0.20763578
190	0.475065905	0.447023685		-0.02804222
191	0.735065687	0.800006335		0.064940649
192	1.15313423	1.093163415		-0.05997081
193	0.91837206	0.920880763		0.002508703
194	0.399513637	0.450446791		0.050933154
195	0.627472242	0.609563344		-0.0179089

196	0.121528668	0.206024724	0.084496057
197	1.267121127	0.340627103	-0.92649402
198	0.609133213	0.281209805	-0.32792341
199	0.387330496	0.39603211	0.008701615
200	0.218995549	0.237908281	0.018912732
201	0.867368192	0.773249234	-0.09411896
202	1.30937951	1.253371934	-0.05600758
203	0.792985544	0.754108146	-0.0388774
204	0.441177619	0.488146262	0.046968643
205	0.710922813	0.71019796	-0.00072485
206	0.895097218	0.862623238	-0.03247398
207	0.596726482	0.577464426	-0.01926206
208	0.583458038	0.568138256	-0.01531978
209	0.14422147	0.175036305	0.030814835
210	0.736017851	0.62897251	-0.10704534
211	0.68530071	0.584639376	-0.10066133
212	0.589968727	0.587782819	-0.00218591
213	0.625570311	0.629852649	0.004282338
214	0.286230981	0.359503958	0.073272977
215	0.395604278	0.370242039	-0.02536224
216	0.747997476	0.742371899	-0.00562558
217	0.863968379	0.793492647	-0.07047573
218	0.352532427	0.322673332	-0.02985909
219	0.150607583	0.140174506	-0.01043308
220	0.213724167	0.160726297	-0.05299787
221	0.329771865	0.323433627	-0.00633824
222	0.126126717	0.072484333	-0.05364238
223	0.296647578	0.269736851	-0.02691073
224	0.306986387	0.337883605	0.030897218
225	0.663314849	0.60647821	-0.05683664
226	0.409588532	0.500623827	0.091035295
227	0.419391128	0.426545627	0.007154499
228	0.055837373	0.059335383	0.00349801
229	0.208428956	0.176631923	-0.03179703
230	0.040588469	0.030373887	-0.01021458
231	0.255916878	0.265949748	0.01003287
232	0.308508357	0.280314202	-0.02819416
233	0.046227048	0.051285347	0.005058298
234	0.258092927	0.268077877	0.009984951
235	0.262242342	0.289622283	0.027379941
236	0.500249856	0.524901681	0.024651825
237	0.288949661	0.284071914	-0.00487775
238	0.078389749	0.117418491	0.039028743
239	0.916827779	1.153448545	0.236620766
240	0.439743334	0.557131714	0.11738838
241	2.01241385	1.763042373	-0.24937148
242	0.976738369	0.805135187	-0.17160318
243	0.653378909	0.631167653	-0.02221126
244	0.292452947	0.296460725	0.004007778

245	0.091991576	0.067511096	-0.02448048
246	0.353157607	0.39967791	0.046520303
247	0.172044721	0.158708495	-0.01333623
248	0.652599568	0.616777202	-0.03582237
249	0.111555378	0.087017786	-0.02453759
250	0.40335708	0.477883434	0.074526353
251	0.262873173	0.131960221	-0.13091295
252	0.218120514	0.118636721	-0.09948379
253	0.248116249	0.156498836	-0.09161741
254	0.294791705	0.330853762	0.036062057
255	0.773597125	0.665108482	-0.10848864
256	1.162850884	1.114963312	-0.04788757
257	0.697642506	0.328700263	-0.36894224
258	0.108683411	0.094620666	-0.01406275
259	0.234467689	0.149041066	-0.08542662
260	0.132956715	0.086768212	-0.0461885
261	0.13293778	0.075520121	-0.05741766
262	0.119751948	0.108464175	-0.01128777
263	0.164228518	0.179130487	0.014901969
264	0.218476313	0.222035728	0.003559415
265	0.226409788	0.192322471	-0.03408732
266	0.21277393	0.227392755	0.014618826
267	0.139052295	0.153113278	0.014060983
268	0.191308127	0.16336357	-0.02794456
269	0.299198353	0.255360677	-0.04383768
270	0.164992063	0.150087465	-0.0149046
271	0.473647153	0.422507261	-0.05113989
272	0.857197697	0.838117811	-0.01907989
273	1.130828234	0.920890655	-0.20993758
274	0.526584567	0.342500886	-0.18408368
275	0.200280566	0.191613588	-0.00866698
276	0.267390766	0.223072333	-0.04431843
277	0.2163677	0.199745206	-0.01662249
278	0.166168605	0.142242026	-0.02392658
279	0.597981449	0.458591641	-0.13938981
280	0.523405267	0.283079941	-0.24032533
281	0.285492629	0.259251887	-0.02624074
282	0.452566806	0.365591993	-0.08697481
283	0.214863906	0.224137945	0.00927404
284	0.262535315	0.285328792	0.022793477
285	0.357583682	0.334716448	-0.02286723
286	0.360407791	0.341637674	-0.01877012
287	0.125317228	0.164318721	0.039001492
288	0.340970122	0.326559078	-0.01441104
289	0.356384672	0.34921814	-0.00716653
290	0.304480601	0.261561712	-0.04291889
291	0.636885336	0.720865222	0.083979886
292	1.3748708	1.267567786	-0.10730301
293	0.386949114	0.256050029	-0.13089908

294	0.633974865	0.554549932	-0.07942493
295	0.612347699	0.514126602	-0.0982211
296	0.637753885	0.588044715	-0.04970917
297	0.339890317	0.26323359	-0.07665673
298	0.55011987	0.388488682	-0.16163119
299	0.222338509	0.343342205	0.121003696
300	0.349690396	0.557266406	0.207576011
301	0.404141708	0.367338281	-0.03680343
302	0.192787329	0.180715831	-0.0120715
303	0.436352715	0.45094491	0.014592194
304	0.994519125	0.914284928	-0.0802342
305	0.979601304	1.001888144	0.02228684
306	0.231291757	0.208517412	-0.02277434
307	0.266241153	0.235952975	-0.03028818
308	0.229488173	0.203897059	-0.02559111
309	0.734284935	0.72907247	-0.00521246
310	0.555379952	0.568310109	0.012930157
311	0.540547187	0.533183443	-0.00736374
312	0.142976023	0.096311232	-0.04666479
313	0.24483658	0.201391915	-0.04344467
314	0.252400936	0.252701396	0.00030046
315	0.200829033	0.17202061	-0.02880842
316	0.074204475	0.095107257	0.020902783
317	0.318710558	0.326955693	0.008245135
318	0.489749333	0.529568953	0.039819621
319	1.6528877	1.523378481	-0.12950922
320	2.331341248	1.960478909	-0.37086234
321	1.192524968	1.047337476	-0.14518749
322	1.036817057	1.070448205	0.033631148
323	0.889070172	0.781750467	-0.10731971
324	0.881610545	0.546248709	-0.33536184
325	0.43428925	0.350070165	-0.08421909
326	0.774988685	0.588440418	-0.18654827
327	0.491554335	0.403964633	-0.0875897
328	0.337598042	0.245618178	-0.09197986
329	0.214367953	0.201130383	-0.01323757
330	0.143759649	0.160308638	0.01654899
331	0.107258994	0.115482741	0.008223747
332	0.494776988	0.362296513	-0.13248048
333	0.40367394	0.364734602	-0.03893934
334	0.407488042	0.332794535	-0.07469351
335	0.823326464	0.685476619	-0.13784985
336	0.591999993	0.476668421	-0.11533157
337	0.833052402	0.706960296	-0.12609211
338	1.00258393	0.921556854	-0.08102708
339	0.333950149	0.411830357	0.077880207
340	0.391097666	0.453128666	0.062031
341	0.325530026	0.362932381	0.037402355
342	0.087697289	0.08716162	-0.00053567

343	0.288281275	0.349632115	0.06135084
344	0.498405976	0.485713238	-0.01269274
345	0.215728131	0.161100112	-0.05462802
346	0.36966243	0.379750125	0.010087695
347	1.215569411	1.120877744	-0.09469167
348	0.078467482	0.062653191	-0.01581429
349	0.323391763	0.181544756	-0.14184701
350	0.049327952	0.024421703	-0.02490625
351	0.24360084	0.222505517	-0.02109532
352	1.273443239	2.302687122	1.029243884
353	0.502150182	0.588685013	0.086534831
354	0.508266853	1.681696841	1.173429988
355	1.09950209	1.578099545	0.478597455
356	0.272973672		

nt number	Δp1 RNA	Δp1 RNA plus NSC
1		
2		
3		
4		
5		
6		
7		
8		
9		
10		
11		
12		
13		
14		
15		
16		
17		
18		
19		
20	-0.08360075	0.303679229
21	0.371424331	0.96639656
22	1.017567327	1.471641697
23	0.810408738	1.391498414
24	0.34168009	1.007722764
25	-0.30416343	0.569291436
26	-0.01995385	0.230397202
27	-0.30884158	0.126037422
28	-0.15183809	0.09882933
29	0.286025063	0.251559754
30	2.194820985	1.836828522
31	2.215979752	2.753503384
32	1.860864655	2.81548387
33	0.414948681	1.446713208
34	2.18980636	2.934094419
35	-0.4423584	0.16875769
36	-0.15884636	0.177888115
37	-0.04475966	0.056861155
38	0.132004122	0.076706939
39	-0.81783988	-0.45750731
40	-0.10305115	-0.1443997
41	-0.42947481	-0.06672733
42	-0.89441488	0.138148108
43	-0.4009147	-0.24263885
44	-0.12868081	-0.28197204
45	-0.07269735	-0.13722395
46	-0.13071083	0.170478007
47	-0.43889	0.063475287
48	-0.50533227	-0.16734434

49	-0.26426546	-0.10796094
50	-0.37008299	0.453654357
51	-0.35229682	-0.4169402
52	0.162326529	0.402205849
53	-0.83092363	-0.78631227
54	0.004754079	-0.11692856
55	0.265056369	-0.34494177
56	-0.03638788	0.10659223
57	0.055118806	-0.34836061
58	0.028439774	-0.46872523
59	0.175230847	0.045988251
60	-0.19692639	-0.39724646
61	-0.14630104	-0.573089
62	-0.26805705	0.333432572
63	-0.45852851	-0.33554542
64	-0.48000471	-0.59894411
65	-0.27472789	-0.37790812
66	-0.22102249	-0.18354432
67	-0.06429255	-0.02363804
68	0.328969251	0.249360841
69	-0.09405389	-0.03658189
70	-0.06237547	-0.18126954
71	-0.18413976	-0.55176684
72	-0.56010968	-0.73928835
73	-0.08107272	-0.23051327
74	0.398398396	0.390168779
75	1.331336813	0.886968166
76	1.590132257	1.761975138
77	1.377816658	1.24977208
78	0.848090736	0.7930813
79	-0.2133501	0.38271963
80	-0.07837959	-0.07548468
81	0.022494204	-0.02235925
82	-0.02607944	-0.00169406
83	0.737120928	0.2604949
84	-0.04837337	-0.12876018
85	-0.28930232	-0.42120848
86	-0.15873759	-0.38854788
87	-0.45087025	-0.50729721
88	-0.33848287	-0.16626964
89	-0.25608792	-0.2281807
90	0.039769964	-0.1072693
91	0.649669473	0.680035779
92	-0.22482564	-0.23289538
93	-0.2131029	-0.13647221
94	-0.13198938	-0.17868426
95	0.10583533	-0.11954104
96	-0.34128819	-0.44972084
97	-0.64594038	-0.32128225

98	0.051327802	-0.18863795
99	-0.15385607	-0.33657066
100	-0.29136958	-0.31982949
101	-0.10152201	-0.32131855
102	0.09182876	0.047620453
103	-0.04830973	-0.13572376
104	0.299892551	0.193603393
105	0.251963153	0.167838025
106	-0.05017129	0.18690911
107	0.142934777	-0.0742543
108	0.181290399	-0.20297099
109	-0.30122409	-0.3597402
110	-0.31720344	-0.23111443
111	-0.45021125	-0.53001321
112	-0.05121277	-0.02231294
113	-0.05318428	-0.23538815
114	-0.38525413	-0.30891273
115	-0.27257051	-0.23387478
116	0.093271577	0.043417982
117	0.438759341	0.513227854
118	0.654544869	0.727650744
119	0.195626045	0.182108227
120	0.012044392	0.029689725
121	-0.25426257	-0.07994443
122	-0.13354914	-0.14489394
123	0.115917887	0.152132799
124	0.127297981	0.095790949
125	-0.16081859	-0.13833657
126	-0.22230682	-0.2583131
127	-0.37780346	-0.70485271
128	-0.26804752	-0.4911522
129	-0.30981814	-0.36108164
130	-0.05291455	-0.39150361
131	0.043285516	-0.30592343
132	0.385294672	0.299164972
133	0.309877892	0.109573706
134	-0.41344481	-0.53258825
135	-0.33347615	-0.67942119
136	-0.02703683	-0.74682177
137	-0.51566075	-0.76721346
138	-0.25249206	-0.68967053
139	-0.20687263	-0.09120864
140	0.132884806	0.069874504
141	-0.13800438	-0.58780702
142	-0.05965592	-0.19558422
143	-0.1161487	-0.05871386
144	-0.17684767	-0.1762856
145	-0.2410771	-0.62964441
146	-0.5144429	-0.5435453

147	-0.0364687	-0.25819211
148	0.164847125	0.03598144
149	0.126380454	-0.15116194
150	-0.10240372	-0.2435411
151	0.00419292	-0.12091614
152	0.061597814	0.054240715
153	0.613695712	0.597321318
154	0.679483251	0.325895912
155	0.716136497	0.419950183
156	1.10223417	0.581727745
157	0.470713151	0.352960983
158	0.047350431	-0.08992483
159	-0.21831792	-0.42171615
160	-0.55307589	-0.80233753
161	-0.16936432	-0.29218375
162	-0.12237566	-0.19501054
163	-0.14982667	-0.573822
164	-0.5813238	-0.68319293
165	0.578993741	0.350669958
166	0.001052561	-0.27535077
167	0.072191989	-0.55886977
168	0.808606445	0.698941151
169	0.764459225	0.342534364
170	1.24264929	1.297940721
171	0.434806862	0.462632483
172	0.005746471	0.041214558
173	-0.20765338	-0.22114176
174	-0.2765775	-0.2996647
175	-0.50868401	-0.33981547
176	-0.45616531	-0.35177315
177	-0.07869779	-0.14952664
178	-0.45427615	-0.2476213
179	-1.73044383	-2.42565527
180	-0.27764565	-0.56607083
181	0.037911549	-0.41370357
182	0.073937167	-0.03811518
183	-1.26054289	-0.62231848
184	1.672647696	1.621836622
185	0.114483687	0.177680991
186	0.18117422	0.026684104
187	-0.23877986	-0.23126468
188	-0.44383622	-0.44094563
189	-0.3417421	-0.60818531
190	0.34794932	0.331513606
191	1.441019863	1.442513349
192	0.576688035	0.639812756
193	0.085911008	0.20413689
194	-0.13729364	-0.05190814
195	-0.21645864	-0.43396547

196	-0.18185281	-0.26216763
197	-0.04255695	-0.3097539
198	0.078477677	-0.05610848
199	-0.15578487	-0.293204
200	0.362563049	0.21956737
201	0.655168479	0.383940927
202	0.636738296	0.580537456
203	0.71968146	0.688595964
204	0.4257013	0.554818274
205	0.447990219	0.570924045
206	-0.27995875	-0.14787633
207	-0.59656524	-0.35342587
208	0.211382206	-0.03576824
209	0.353343403	0.246738728
210	0.352064679	0.21816201
211	0.133677556	0.08493713
212	0.200858083	-0.00049282
213	0.116669213	-0.75659181
214	0.075395835	-0.02605714
215	0.506406292	0.444265628
216	0.947851655	1.089097403
217	0.277230499	0.26353862
218	-0.15601546	-0.35426676
219	-0.43371295	-0.6608417
220	-0.12244193	-0.37279513
221	-0.06416701	-0.42332446
222	0.047476642	-0.40736989
223	0.706093219	-0.49400762
224	0.03370155	-0.19280492
225	0.509965883	0.572261632
226	0.366155859	-0.18185907
227	-0.08314188	-0.18641896
228	-0.05213096	-0.08001868
229	-0.2022043	-0.02519411
230	-0.07172596	0.111404049
231	-0.3627693	-0.31701603
232	-0.25766741	-0.2618578
233	-0.35972648	-0.42669789
234	0.082513716	0.272452567
235	0.356731152	0.300551358
236	-0.14062371	-0.16339403
237	-0.29462903	-0.10694092
238	-1.88695705	-1.38922549
239	0.44474826	0.195711508
240	2.174752967	2.375744479
241	1.442658414	1.690722938
242	0.71959906	0.706716486
243	-0.22807848	-0.41307272
244	-0.15565987	-0.42429669

245	-0.24217973	-0.7321558
246	-0.54921787	-0.46480563
247	1.091794191	1.382170797
248	-0.45279823	-0.40821287
249	-0.70674269	-0.72400505
250	-0.72832116	-1.07947183
251	0.027806546	0.030905294
252	-0.38542007	-0.52064184
253	-0.31973197	-0.31879855
254	-0.09088499	-0.21868354
255	0.49703153	0.437244472
256	1.736757376	2.187857246
257	-0.46634467	-0.41455818
258	-0.04675007	-0.1512673
259	-0.29266193	-0.38761586
260	-0.0905796	-0.24526946
261	0.102593532	-0.00543268
262	-0.33803918	-0.43731858
263	-0.14000756	-0.32499546
264	-0.42306127	-0.57785646
265	-0.2211777	-0.20035433
266	-0.32797613	-0.36360618
267	-0.95487379	-0.77632142
268	-0.33639645	-0.48017212
269	-0.15075217	-0.16394369
270	-0.25888947	-0.06601708
271	0.340675415	0.173476106
272	-0.19711815	-0.02113201
273	1.592359568	2.03731172
274	-0.33280282	-0.46682978
275	-0.19130799	-0.28010036
276	-0.22162109	-0.16361738
277	0.156756642	0.142343219
278	-0.39503429	-0.2452399
279	-0.10601335	-0.01501704
280	0.929321411	0.69183689
281	-0.15874025	0.032739825
282	-0.45882847	-0.35029225
283	-0.04409818	0.109697115
284	-0.40734972	-0.52120268
285	0.181120768	-0.18572111
286	0.056831752	-0.11530686
287	-0.1034458	-0.12737609
288	-0.03499416	0.142047685
289	-0.59810704	-0.85209862
290	1.436580295	2.042309091
291	0.869172896	1.165919997
292	0.461454505	1.008088658
293	0.619701322	-0.0222044

294	0.306797506	-0.40817362
295	0.489022771	0.882350236
296	1.082330726	1.565346966
297	-0.20597746	-0.02614104
298	0.238330959	0.336803164
299	-0.21191393	-0.2070156
300	-0.36335651	-1.15257912
301	-0.10044401	-1.08228236
302	1.14106728	0.782032949
303	-0.21651475	-0.25690702
304	-0.01293938	0.581604436
305	0.88714909	0.801028352
306	0.335833876	0.363866661
307	0.398222942	0.813503415
308	0.575981586	0.661281916
309	0.417040108	0.372626227
310	-0.01893249	-0.86042635
311	-0.49647162	-0.77551645
312	-0.1561927	0.046420427
313	0.393025111	0.462591162
314	-0.013182	0.05777717
315	0.241359997	-0.08116665
316	-0.41336527	0.074127047
317	0.377739169	-0.03440924
318	0.267867171	-0.1200703
319	0.10185529	0.418470121
320	-0.1997564	0.046391619
321	0.24979876	-0.04815622
322	0.328902385	-0.0106544
323	-0.19238682	0.068491235
324	-0.1502415	-0.84357131
325	0.248903418	0.327922351
326	0.861038107	0.60613896
327	0.363329587	0.321267771
328	0.917878718	0.436441275
329	0.066946462	-0.57003801
330	0.30396811	0.448477279
331	0.344431845	0.62970429
332	0.02203193	0.139808398
333	-0.13929535	0.486316226
334	0.343472866	1.450316639
335	1.017602908	4.675572182
336		0.540536446
337		6.787912744
